# Does Passive Leg Activity Influence Oxygen Saturation and Activity in Sedentary Elderly Adults?

**DOI:** 10.1100/tsw.2006.199

**Published:** 2006-08-31

**Authors:** Eli Carmeli, Gadi Bartur, Sara Peleg, Shemuel Barchad, Jean-Jacques Vatine

**Affiliations:** ^1^ Department of Physical Therapy, Stanley Steyer School of Health Professions, Tel Aviv University, Ramat Aviv 69978, Israel; ^2^ Reut Medical Center, Yad Elyahu, Rechasim, Israel; ^3^ Neve Ram Residential Care Center, Rechasim, Israel

**Keywords:** intellectual disability, positioning, oxygen saturation, long-term care, Israel

## Abstract

This study aimed to investigate whether any physiological changes might have a clinically significant effect on function in sedentary, institutionalized, older adults treated by a passive training program. A total of 18 subjects (mean age 60.7 ± 3.4) with intellectual disability (ID) participated. We measured SpO2 (arterial oxygen saturation) before, during, and after passive training, and used Barthel Index to measure daily living activities. The general trend indicated that inactive people with ID evidenced a continual increase in SpO2% levels and some functional gains during passive treatment, with superiority to manual passive treatment compared to mechanical active passive training. For current clinical practice, most sedentary patients who experience clinically significant deconditioning and desaturation can benefit from passive treatment.

## INTRODUCTION

Physical deconditioning, bed rest, and a passive lifestyle are common among aged people with intellectual disability (ID). Although some forms of passive activities have been shown to be beneficial for cerebral palsy children, people with spinal cord injury[1], and stroke patients, a “pure” passive training program has never been introduced for inactive older adults with ID living in residential care. With increasing life expectancy and improving care and awareness, the numbers of aged persons with ID is steadily increasing. The major reasons for increased life expectancy are attributed to improvements in medical technology and in the quality of health and social care[2].

Currently, care staff often restrain residents to avoid aspiration, prevent complications such as pressure sores and contractures, support and stabilize body segments, improve circulation, prevent edema, modify muscle tone, provide appropriate sensory information, increase spatial awareness, and enhance comfort[3,4]. The effect of inactivity was investigated in a cross-sectional study conducted at an in-patient facility, on residents aged between 20 and 90 years. The results showed a clinically important and highly significant decline of oxygen tension (SpO2%) with a minor effect on blood gas values[5].

However, the effects of positional restraint on heart rate and oxygen saturation that was used in 18 individuals (seated, unrestrained) resulted with no clinical implications on heart rate or oxygen saturation[6]. Reay et al.[7] reported that positional restraint induces peripheral oxygen saturation and heart rate decline, and produced a mean recovery time significantly prolonged[7].

In fact, there are no studies that measured arterial oxygen saturation (SpO2%) in sedentary older adults, sitting or lying in bed, while receiving a passive range of motion (ROM) treatment. Therefore, the following study was conducted to determine whether any physiological changes might have a clinically significant effect on function in sedentary, institutionalized, older adults treated by a passive training program involving leg cycling. We hypothesized that passive exercise may contribute to improved functional outcomes.

## METHODS

This study was designed to answer the question about the effect of a passive leg cycling program on sedentary older adults with ID. The null hypothesis was that a structural passive exercise program has little or no effect in improving arterial oxygen saturation (SpO2%) and basic activities of daily living (ADL).

Subjects were recruited from permanent residents living in a residential care center, “Neve Ram” (Rechasim) in the north of Israel. The subjects were 18 individuals diagnosed with a mild ID level. The range of the general IQ level was from 57.22–64.70. Age range was 55–72 (mean age 57 ± 5.2). Each subject's primary care physician approved participation in the study. A total of 18 subjects (10 females and 8 males) participated in this study. Group A included 10 sedentary adults (5 females and 5 males), aged 55–65; group B included 8 sedentary adults (5 females and 3 males), aged (mean age 60.7 ± 3.4) and gender matched. “Sedenteriness” was determined by nonactive lifestyle >50% of “awakening hours” (i.e., 07:00 AM through 07:00 PM).

Persons with uncontrolled blood pressure, metabolic disorder (i.e., diabetes, renal failure), and severe chest wall deformity were excluded. Inclusion criteria for the trial were the following: (1) living in a residential care center for at least 5 years; (2) moderate to severe intellectual deficits, i.e., less than 56; (3) oxygen dependence; (4) required moderate to maximum assistance for most of the daily activities; and (5) their lifestyle was characterized as “sedentary”.

This study was approved by the Ministry of Social Affairs and written informed consent was obtained from all legal guardians.

### Measures

After baseline assessments, the subjects were randomly assigned to two groups through the use of a random number method. Outcome assessments were performed by the principal investigator blinded to treatment assignment. A standard and routine noninvasive monitoring of arterial blood oxygen saturation was measured by a digit finger oximeter (Smiths Medical, Watford, Herts, U.K.) before, during, and after training with a mechanical Active Passive Trainer (APT, Tzora Health Care Products, Israel) and manual passive treatment. The leg APT was adjusted to accommodate the subject's ROM at the hip and knee, and was performed on their bed.

Oximetry is a useful monitor of the subject's cardiorespiratory system. The pulse oximetry (SpO2 levels and heart rate) measured the average amount of oxygen bound to each haemoglobin molecule as well as the heart rate every 15 sec.

### Physiological Assessments

As a baseline and for monitoring purposes, arterial oxygen saturation (SpO2%), heart rate (1 min), blood pressure (mmHg), and respiration rate were recorded before, right after, and 30 min after each training session.

## Barthel Index

The original Barthel activity of daily living index (BI)[8] consists of 14 tasks(task no 15 is stipulated and should be applied only 'if not walking'): tasks 1–9, the self-care index, including control of bladder and bowel sphincters, have a total possible score of 53. Tasks 10–14, the mobility index, have a total possible score of 47. The groups of tasks combined make up the total BI with a total possible score of 100. Outcome was categorized primarily based on the BI as: excellent (80–100), good (60–79), moderate (40–59), and poor (0–39).

## Passive Training Program

A mechanical and a manual passive leg cycling program was designed to improve oxygen saturation, improve ROM of the lower extremity joints, encourage more use of the limbs, and improve basic ADL. For monitoring purposes, arterial oxygen saturation ($SpO_2$%), heart pulse (1 min), blood pressure (mmHg), and respiration rate were recorded before, during, and after each training session. Participants were exercised on the APT or manually 4–5 times per week, for 12 consecutive weeks. The passive training program always took place between 09:30 and 11:30 AM, indoors, under controlled conditions (23ºC, 40% humidity). A therapist stood by the APT (group A) as a safety precaution in case of unexpected discomfort.

## Group A

The mechanical treatment was conducted by APT. Prior to each session, a prolonged and progressive stretching to Achilles tendon, hamstrings, and quadriceps muscles was performed for 5 min. Then, an unloaded level of continuous passive pedaling was undertaken for 5 min. The passive pedaling consisted of individually prescribed sessions (50–70 rpm with 0 W), as tolerated pain free, and gradually increased for as long as 20 min, as endurance improved.

## Group B

The manual passive treatment was conducted by two trained physical therapists using the same protocol. Treatment time followed the same framework as group A, i.e., started initially for 5 min, then gradually increased up to 20 min. The passive intervention was conducted according to the proprioceptive neuromuscular facilitation (PNF) patterns (known as D1 and D2) for both upper and lower extremities. Ten consecutive and slow amplitude repetitions (× 5) were performed on one side of the upper body, then on the opposite side in each major joint (shoulder, elbow, and wrist), then to the lower limbs (hip, knee, and ankle). No trunk movements were applied.

## Statistical Analysis

All statistical analyses were performed using a commercial software package (SPSS version 11, SPSS Inc, Chicago, IL). Chi square tests for cross-tabulation tables and Student's t-test were used to compare the $SpO_2$%, functional outcomes, and basic ADL of the two groups (A and B) at initial assessment. Within-group differences from the baseline to post-training measurements were also assessed using Student’s t-test (two-tailed) for repeated measures. Standard procedures were used for all variables to calculate mean ± SD, and correlation coefficient (Pearson r). A two-factor ANOVA (time and group) was used to determine the effect of passive training on $SpO_2$% and basic ADL. Statistical significance was accepted at an alpha level < 0.05.

## RESULTS

All 18 subjects completed the 12-week program. All subjects received passive training program as planned. The passive treatments were protocol driven and included progression of duration. Each subject received an average of 51.3 ± 3.2 visits, and the average duration of a visit was 16.4 ± 1.7 min. The general trend of the results indicated that inactive people with ID evidenced a continual increase in SpO2% levels and some functional gains during passive treatment.

Barthel index (BI) scores and SpO2% rates were examined. In order to examine the relationships among gender, a repeated measures ANOVA was conducted using BI and SpO2% levels before and after the passive training program as the dependent variable. Table 1 contains the basic ADL outcomes measured by BI scores and SpO2% levels at T1 and T2 (12 weeks later).

**TABLE 1**
**BI Scores and SpO2% Levels**

| | Group A, APT | | Group B, Manual Passive Move | |
|---|---|---|---|---|
| | Male (n = 5) T1/T2 | Female (n = 5) T1/T2 | Male (n = 3) T1/T2 | Female (n = 5) T1/T2 |
| 1. Drinking from a cup | 16/20 | 20/20 | 8/12 | 8/12 |
| 2. Eating | 0/0 | 0/24 | 0/12 | 0/24 |
| 3. Dressing upper body | 0/0 | 0/0 | 0/12 | 0/16 |
| 4. Dressing lower body | 0/0 | 0/20 | 0/12 | 4/16 |
| 5. Putting on brace or show | 0/0 | 0/8 | 0/8 | 0/10 |
| 6. Grooming | 0/0 | 0/15 | 0/15 | 0/20 |
| 7. Washing or bathing | 0/0 | 0/0 | 0/0 | 0/0 |
| 8. Controlling urination | 25/25 | 25/25 | 15/25 | 15/25 |
| 9. Controlling bowel movements | 25/25 | 25/25 | 15/25 | 15/25 |
| 10. Getting in and out of chair | 0/21 | 7/21 | 14/21 | 7/21 |
| 11. Getting on and off toilet | 12/15 | 12/15 | 6/9 | 12/15 |
| 12. Getting in and out of tub or shower | 0/0 | 0/1 | 0/3 | 0/1 |
| 13. Walking 25 m on the level | NA | NA | NA | NA |
| 14. Walking up/down 6 stairs | NA | NA | NA | NA |
| 15. If not walking: propelling or pushing wheelchair | 15/15 | 15/15 | 10/15 | 20/25 |
| SaO2% | | | | |
| Before | 90/91 | 90/91 | 90/90 | 89/90 |
| After | 92/93 | 91/92 | 94/95 | 94/95 |
| 30 min after | 91/92 | 91/91 | 92/92 | 92/92 |

The overall effect of the intervention compared between pre- and post-treatment was highly significant ($p < 0.005$) by a single multivariate ANOVA. Both groups demonstrated improvement from baseline to 12 weeks in most BI tasks, yet only group B showed a significant improvement. Group A did not show gains in five tasks (3, 7, 8, 9, 15), whereas group B achieved greater gains in 10 out of 13 tasks. In both groups, the females achieved higher scores than males following 12 weeks of the intervention.

Table 2 contains the summary of the repeated measures ANOVA. In both groups, the within-subjects (A and B) results indicated a significant change between pre- and postpassive training. The independent

**TABLE 2**
**Summary for Single-Factor Repeated Measures ANOVA: SpO2% in Three Phases (n = 18)**

| Source of Variance | *df* | SS | MS | F | *p* |
|---|---|---|---|---|---|
| Subjects (S) | 17 (n-1) | 5208 | 652 | 88.75 | NA |
| SaO2% (A) | 2 (*k*-1) | 1453.72 | 724 | 102.33 | <0.05 |
| Error (A × S) | 34 (n-1) (k-1) | 234.02 | 14.64 | | |
| Total | 53 (nk-1) | 6895.75 | | | |

variable was the three phases of the passive movements, i.e., three levels (k = 3), before, right after, and 30 min later. Thus, this warrants a repeated measures design, where each subject's SpO2% is tested in each phase of the intervention. We concluded that SpO2% was related to the time it was measured (i.e., before, right after, 30 min later). A significant increase in SpO2% was found in group B during passive treatment given during the last 2 weeks of the program compared with the SpO2% levels of the first 2 weeks ($F4, 152 = 8.26$, $p < 0.05$). Whereas, in group A, the SpO2% levels rose during the first week and remained constant until the treatment program concluded. SpO2% levels of the subjects rose during passive treatment in group B ($p < 0.005$) more than in group A ($p$ = NS).

Adverse events were monitored and reported according to a protocol approved by the Ministry of Social Affairs. There were no musculoskeletal pains or cardiovascular disorders.

## DISCUSSION

This study is unique in that it is the first geriatric study to our knowledge to describe the physiologic and functional changes, and to evaluate the safety of using a mechanical trainer (APT), in persons with intellectual disability (ID). Since the investigated subjects spend most of their time in bed or on a chair for meals and showers, we hypothesized that a passive training program would result in clinically important changes. To do so, we monitored the peripheral oxygen saturation at rest, during passive exercise, and during recovery.

There is general acknowledgment to the fact that passive treatment, manual as well as mechanical, is particularly important in preventing secondary conditions for active[9,10] and inactive people[11]. This structural study first supports it, but more importantly, compares the effectiveness between two treatment approaches: manual passive treatment vs. mechanical passive treatment. When BI scores and SaO2% levels were compared to baseline measurements, both immediate and cumulative improvement in oxygen as well as in function were seen in both groups. Furthermore, this study was able to identify which passive intervention had greater efficacy. The superiority of the manual treatment compared with the mechanical was consistent in all the investigated parameters. There has been a number of studies reporting the effectiveness of passive treatment in sedentary patients, and the effect of remobilization on SpO2%[6,7]. Our findings also support those of Blom et al.[5], who demonstrated improvement of arterial oxygen tension and saturation in hospital patients following passive treatment. More over, Sato et al.[10]. demonstrated that even young sprinters could slow heart rate responses at the onset of passive movement as compared with untrained subjects.

The effects of temporary inactivity such as “positional restraint” produced a mean recovery time that was significantly prolonged. Chang et al.[11] provided evidence of improved short-term ventilatory function following a passive neurophysiological facilitation (utilizing PNF techniques), supporting its use in the management of high-dependency neurological patients. Doering et al.[9] showed that active as well as passive exercises are followed by an increase of cerebral blood flow velocity, and they attributed the increase of hemodynamic and metabolism to autoregulative mechanisms. Bell et al.[12] concluded that passive exercise on an upright bicycle contributes to both the neural and metabolic influences toward the physiological adjustments observed[12].

The differences between manual passive treatments compared to mechanical passive treatments are probably due to the accuracy of the first one. The manual therapist can adjust his/her maneuvers to the patient's position and overall comfort, and to modify the maneuvers to the general tolerance. The therapist providing the manual passive treatment was capable of “listening” to the soft tissue resistance and resilience throughout the passive range of the joint movements. We may have underestimated the behavior of the patient during the treatment. Perhaps those patients who received manual passive treatment changed their position during the treatment more often and that by itself could increase chest movements, and therefore their lung ventilation was more adequate and profound. Although mechanical passive treatment, which increases SpO2% and functional abilities to some extent, may seem cost effective and easy to apply, it might not provide significant and long-term benefits achieved by manual treatment.

What are the implications of this study? For current clinical practice, most sedentary patients who experience clinically significant deconditioning and desaturation can benefit from passive treatment.
